# Comparison of the accuracy of neutrophil CD64, procalcitonin, and C-reactive protein for sepsis identification: a systematic review and meta-analysis

**DOI:** 10.1186/s13613-018-0479-2

**Published:** 2019-01-08

**Authors:** Chun-Fu Yeh, Chin-Chieh Wu, Su-Hsun Liu, Kuan-Fu Chen

**Affiliations:** 1 Division of Infectious Diseases, Department of Internal Medicine, Chang Gung Memorial Hospital, Linkou, Taiwan; 2 Graduate Institute of Clinical Medical Sciences, College of Medicine, Chang Gung University, Taoyuan, Taiwan; 3 Department of Emergency Medicine, Chang Gung Memorial Hospital, Keelung, Taiwan; 4 Health Management Center, Far Eastern Memorial Hospital, New Taipei, Taiwan; 5 International Health Program, National Yang Ming University, Taipei, Taiwan; 6 Clinical Informatics and Medical Statistics Research Center, Chang Gung University, 5 Fu-Shin Street, Gueishan District, Taoyuan, 333 Taiwan; 7 Community Medicine Research Center, Chang Gung Memorial Hospital, Keelung, Taiwan

**Keywords:** Sepsis, Biomarker, Neutrophil CD64, Meta-analysis

## Abstract

**Background:**

Neutrophil CD64 is widely described as an accurate biomarker for the diagnosis of infection in patients with septic syndrome. We performed a systematic review and meta-analysis to evaluate the diagnostic accuracy of neutrophil CD64, comparing it with C-reactive protein (CRP) and procalcitonin (PCT) for the diagnosis of infection in adult patients with septic syndrome, based on sepsis-2 criteria. We searched the PubMed and Embase databases and Google Scholar. Original studies reporting the performance of neutrophil CD64 for sepsis diagnosis in adult patients were retained. The pooled sensitivity, specificity, diagnostic odds ratio (DOR), and hierarchical summary receiver operating characteristic (SROC) curve were calculated.

**Results:**

We included 14 studies (2471 patients) from 2006 to 2017 in the meta-analysis. The pooled sensitivity and specificity of neutrophil CD64 for diagnosing infection in adult patients with septic syndrome were 0.87 (95% CI 0.80–0.92) and 0.89 (95% CI 0.82–0.93), respectively. The area under the SROC curve and the DOR were 0.94 (95% CI 0.92–0.96) and 53 (95% CI 22–128), respectively. There was significant heterogeneity between the studies included. Subgroup analyses showed that this heterogeneity was due to differences in sample size and the proportions of patients with sepsis included in the studies. Six studies (927 patients) compared neutrophil CD64 and CRP determinations, and six studies (744 patients) compared neutrophil CD64 and PCT determinations. The area under the SROC curve was larger for neutrophil CD64 than for CRP (0.89 [95% CI 0.87–0.92] vs. 0.84 [95% CI 0.80–0.88], *P* < 0.05) or PCT (0.89 [95% CI 0.84–0.95] vs. 0.84 [95% CI 0.79–0.89], *P* < 0.05).

**Conclusions:**

In adult patients with septic syndrome, neutrophil CD64 levels are an excellent biomarker with moderate accuracy outperforming both CRP and PCT determinations.

**Electronic supplementary material:**

The online version of this article (10.1186/s13613-018-0479-2) contains supplementary material, which is available to authorized users.

## Background

Sepsis is a life-threatening disease with a mortality of 18% to 45% in critically ill patients [1, 2]. Despite progress in the clinical guidelines for treating sepsis, early identification and the use of broad-spectrum antibiotics remain the cornerstone of treatment. A missed identification of sepsis delays treatment, increasing the risk of death [3]. By contrast, the overuse of antimicrobial agents in patients without sepsis leads to antibiotic resistance. The accurate identification of sepsis is, therefore, crucial, to improve clinical outcomes and reduce medical costs. A new definition of sepsis (sepsis-3) has been recently proposed, based on the detection of infection together with organ dysfunction and based on sequential organ failure assessment score (SOFA score) [4]. However, it remains difficult to detect the onset of sepsis-3, as this early identification is still based on clinical symptoms and signs including fever, dyspnea, tachycardia, leukocytosis/leukopenia, or bandemia in patients with sepsis according sepsis-2 criteria [5]. A biomarker is therefore urgently required to improve the early diagnosis of infection in patients with septic syndrome.

Many studies have identified neutrophil cluster of differentiation 64 (CD64) as a candidate biomarker for bacterial infection and sepsis [6–8]. CD64 is an Fcγ receptor expressed principally on monocytes and, to a much lesser extent, on resting polymorphonuclear leukocytes (PMNs). Bacterial infection or sepsis leads to an increase in CD64 expression on activated PMNs [6]. CD64 levels on the surface of PMNs can be evaluated with a flow cytometer and a Leuko64 kit (Trillium Diagnostics LLC., Brewer, Maine, USA) or by in-house staining with fluorochrome-labeled anti-CD64 antibodies. Neutrophil CD64 is now considered a candidate biomarker of sepsis suitable for use in clinical practice.

Two meta-analyses have demonstrated the diagnostic power of neutrophil CD64 for bacterial infection. They reported similar areas under the SROC curve of 0.94 and 0.92, resulting in sensitivities of 0.79 and 0.76 and specificities of 0.91 and 0.85, respectively [9, 10]. A similar result was obtained for the identification, with neutrophil CD64, of sepsis based on sepsis-2 criteria in critically ill patients, with a sensitivity, specificity, and area under the SROC curve of 0.76 (95% confidence interval [CI] 0.73–0.78), 0.85 (95% CI 0.82–0.87), and 0.95, respectively [11]. Despite the high area under the SROC curve for the diagnosis of bacterial infection and sepsis with neutrophil CD64, this method has been reported to have a relatively low sensitivity, with reported values ranging widely, from 0.66 to 0.96. No meta-analysis has ever compared the diagnostic performance of neutrophil CD64 with that of other widely used biomarkers, such as C-reactive protein (CRP) and procalcitonin (PCT). We evaluated the accuracy of neutrophil CD64 as a biomarker for diagnosing infection in adult patients with septic syndrome based on sepsis-2 criteria, by performing a systematic literature review and a meta-analysis, comparing the diagnostic value of neutrophil CD64 with that of CRP and PCT.

## Materials and methods

### Data source

The systematic review and meta-analysis were performed according to the Preferred Reporting Items for Systematic Reviews and Meta-Analyses guidelines [12]. We searched the PubMed and Embase databases for studies on the diagnostic accuracy of neutrophil CD64 for sepsis published before July 2017. We used the following keywords: ((“Systemic Inflammatory Response Syndrome” OR “SIRS”) AND “Sepsis”) AND (“Early Diagnosis” OR “Diagnosis”) AND (“CD64” OR “neutrophil CD64”) AND “adult” in PubMed and (sensitivity OR diagnostic AND accuracy:link OR diagnostic AND (“sepsis”/exp OR sepsis) AND (“CD64” OR neutrophil CD64) AND [english]/lim) in EMBASE. We also searched Google Scholar with the following keywords: “diagnostic”, “CD64”, “sepsis”, and “adult”. In addition, we checked the reference lists of each of the primary studies to identify additional publications.

### Study eligibility

Studies were included if they were (1) original, (2) dealt with the diagnostic accuracy of neutrophil CD64 for sepsis (3), included adult patients, and (4) written in English. Studies were excluded if they met any of the following criteria: (1) insufficient information to construct a 2 × 2 contingency table; (2) a duplicated study; (3) prognosis based on the prediction of mortality from sepsis; and (4) a review article, conference paper, or case report. The eligibility of studies was independently evaluated by two authors (Chun-Fu Yeh and Chin-Chieh Wu).

### Data extraction

These two authors independently extracted data from each study, including year of publication, country of origin, study design, source of patients (emergency departments [EDs] or intensive care units [ICUs]), sample size, case and control numbers, assay methods, diagnostic cutoff points, sensitivity, and specificity. These data were used to construct a 2 × 2 contingency table. Further information was obtained by sending an e-mail request to the original author if the necessary information was not available.

### Quality Assessment

Study methodology was assessed with the Quality Assessment of Diagnostic Accuracy Studies-2 (QUADAS-2) checklist [13]. QUADAS-2 was used to assess the risk of bias and applicability in four key areas: patient selection, index test, reference standard, and flow and timing. The two authors performed quality and risk of bias assessments independently. Discrepancies between their findings concerning study eligibility, data extraction, and study quality were resolved at consensus meetings.

### Statistical analyses

All statistical analyses were performed with the Midas module in Stata 13.1 (Stata Corporation, College Station, TX, USA) and the mada package in R (version 3.1.3, R Foundation for Statistical Computing). Interobserver agreement between the two authors concerning study eligibility was assessed by calculating Cohen’s kappa statistic. A graphical display of the quality of the studies included was generated with the Midas and QUADAS modules in Stata. All statistical tests were two-tailed, and values of *P* < 0.05 were considered significant.

A hierarchical summary receiver operating characteristic (HSROC) model, as proposed by Rutter and Gatsonis [14], was used to calculate the pooled sensitivity and specificity, the diagnostic odds ratio (DOR), and the area under the summary receiver operating characteristic (AUHSROC) curve of the studies included. We also constructed the corresponding SROC curve, by plotting the sensitivity and specificity of the included studies (as a pair) in the receiver operating characteristic space, and we then calculated the area under the curve [15, 16].

Threshold and nonthreshold effects of heterogeneity in the included studies were assessed. The threshold effect was evaluated by determining Spearman’s correlation coefficient (*ρ*) for the relationship between the logarithms of sensitivity and 1-specificity and from visual inspection of the SROC curve. The nonthreshold effect was calculated with Chi-squared (*χ*^2^) tests, Cochran’s *Q* test, and the *I*^2^ metric. Heterogeneity between the studies was considered to be present if *I*^2^ was greater than 50%. We then performed a univariable metaregression analysis with a bivariate binomial mixed-effect model and subgroup analysis, to explore the sources of heterogeneity. The covariates included source of patients, assay methods, sample size, proportions of patients with sepsis, and country of origin. Publication bias was assessed with Deeks’ funnel plots.

We compared neutrophil CD64 with CRP and PCT, by selecting the studies directly comparing CD64 with CRP and/or PCT from the list of studies included. The area under each SROC curve was calculated. The areas under the two SROC curves were compared as described by Rosman and Korsten [17].

## Results

### Study selection

**Fig. 1:**
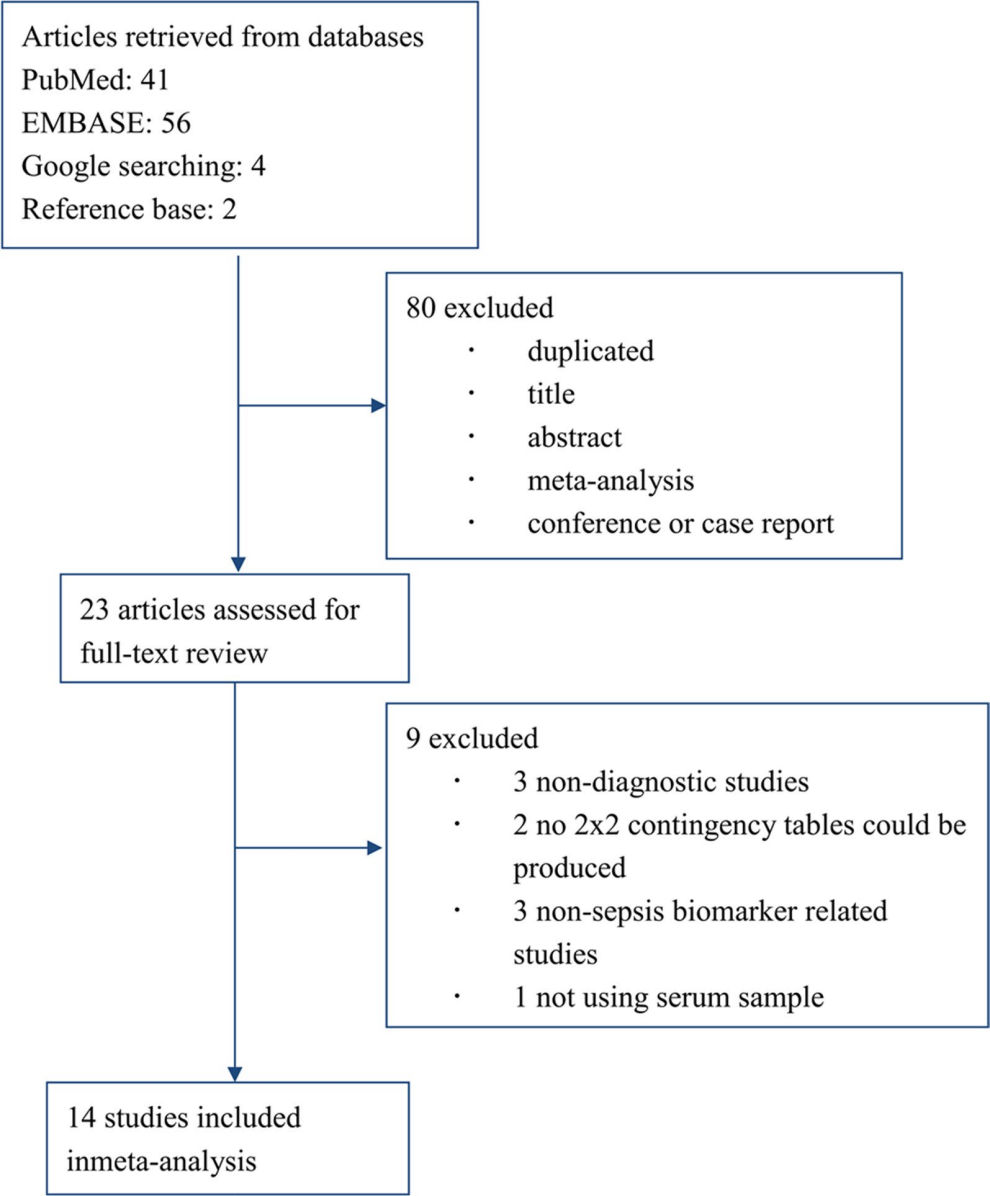
Flowchart of study selection, inclusion, and exclusion for the meta-analysis of CD64 for diagnosis of sepsis

We retained 103 abstracts in total: 56 from Embase, 41 from PubMed, 4 from Google Scholar, and 2 from the reference lists of related articles (Fig. 1). We excluded 80 articles during the initial screening. Twenty-three articles were subjected to further review, and nine articles were excluded (3 were not diagnostic studies, 2 did insufficient information for the construction of a 2 × 2 contingency table, 3 were not relevant to the use of neutrophil CD64 as a sepsis biomarker, and 1 did not use the serum sample). In total, 14 studies were retained for the final analysis [18–31].

### Study characteristics

The features of the 14 selected studies are presented in Table 1. The studies were published from 2006 to 2017: 10 were prospective [18, 19, 21–24, 26, 27, 29, 31], two were retrospective [20, 28], and the study design was not clearly described in the other two [25, 30]. Eleven studies included selected patients from ICUs [19–21, 23–30], and three studies included patients from EDs [18, 22, 31]. Thirteen studies used the 2001 American College of Chest Physicians/Society of Critical Care Medicine criteria for sepsis diagnosis [19–31], and one study used the clinical infection/sepsis score developed from the same criteria [18].

**Table 1:** Main characteristics of the studies selected for the meta-analysis

References	Country	Study design	Mean age (years) total or case/control	Patient source	Sepsis/control (*n*)	Total (*n*)	Proportion of patients with sepsis (%)	Assay method	Cutoff	Sensitivity, specificity	TP (*n*)	FP (*n*)	FN (*n*)	TN (*n*)
Davis et al. [18]	USA	Prosp	–	EDs	38/62	100	38.0	In-house	2000 MESF	0.88, 0.71	33	18	5	44
Livaditi et al. [19]	Greece	Prosp	–	ICUs	47/12	59	79.7	In-house	2566 mol/cell	0.95, 1.00	44	0	3	12
Cardelli et al. [20]	Italy	Retro	63	ICUs	52/60	112	46.4	In-house	2398 mol/cell	0.96, 0.95	50	3	2	57
Hsu et al. [21]	China	Prosp	67/80	ICUs	55/11	66	83.3	In-house	4300 mol/cell	0.89, 0.96	49	1	6	10
Gamez-Diaz et al. [22]	Colombia	Prosp	–	EDs	404/206	610	66.2	Leuko64 kit	1.7 MESF	0.66, 0.65	266	73	138	133
Gibot et al. [23]	France	Prosp	61/60	ICUs	154/146	300	51.3	In-house	1.62	0.84, 0.95	130	7	24	139
Gros et al. [24]	France	Prosp	61/58	ICUs	148/145	293	50.5	Leuko64 kit	2.2	0.63, 0.89	93	16	55	129
Gerrits et al. [25]	Netherlands	Not described	74/69	ICUs	25/19	44	56.8	Leuko64 kit	1.66	1.00, 0.95	25	1	0	18
Dimoula et al. [26]	Belgium	Prosp	61/58	ICUs	103/365	468	22.0	In-house	230 MFI	0.89, 0.87	92	47	11	318
Righi et al. [27]	Italy	Prosp	59/58	ICUs	61/32	93	65.6	In-house	2000 ABC	0.90, 0.97	55	1	6	31
Godnic et al. [28]	Germany	Retro	–	ICUs	40/7	47	85.1	Leuko64 kit	1.95	0.76, 0.75	30	1	10	6
Bauer et al. [29]	USA	Prosp	66/59	ICUs	110/86	196	54.8	In-house	1040.5 mol/cell	0.76, 0.77	84	20	26	66
Muzlovic et al. [30]	Slovenia	–	–	ICUs	25/7	32	78.1	Leuko64 kit	1.58	1.00, 0.86	25	1	0	6
Tan et al. [31]	Malaysia	Prosp	–	EDs	42/9	51	82.4	In-house	45 ABC	0.81, 0.89	34	1	8	8

− not available, *ABC* antibody-binding capacity, *EDs* emergency departments, *FCM* flow cytometry, *FN* false negative, *FP* false positive, *ICUs* intensive care units, *MFI* median fluorescence intensity, *Prosp* prospective, *Retro* retrospective, *TN* true negative, *TP* true positive

The final analysis included 2471 patients: 1304 patients with sepsis (52.8%) and 1167 controls (47.2%). These 2471 patients comprised 1710 (69.2%) patients from ICUs and 761 (30.8%) from EDs. The percentage of patients with sepsis ranged from 22.0% to 85.1%.

### CD64 assay

In-house flow cytometry assays were performed to determine neutrophil CD64 levels in nine studies [18–21, 23, 26, 27, 29, 31], whereas Leuko64 kits were used in the other five studies [22, 24, 25, 28, 30]. The cutoff values for CD64 levels differed between studies (Table 1).

### Quality Assessment

The QUADAS-2 results are presented in Table 2 and Additional file 1: Figure S1. Eight studies met the reference standard criterion [18, 21–24, 26, 27, 29]. Twelve met the flow and timing criterion [18–21, 23–28, 30, 31]. For patient selection, six studies were considered to have a low risk of bias [23, 24, 26, 27, 29, 31], three had a high risk of bias due to their case–control design [19, 20, 22], and the details of patient selection were not clearly reported in five studies [18, 21, 25, 28, 30]. None of the studies met the index test criterion in the risk of bias analysis, because no prespecified threshold was used for the index test. For applicability, all the studies met the criterion for low risk.

**Table 2:**
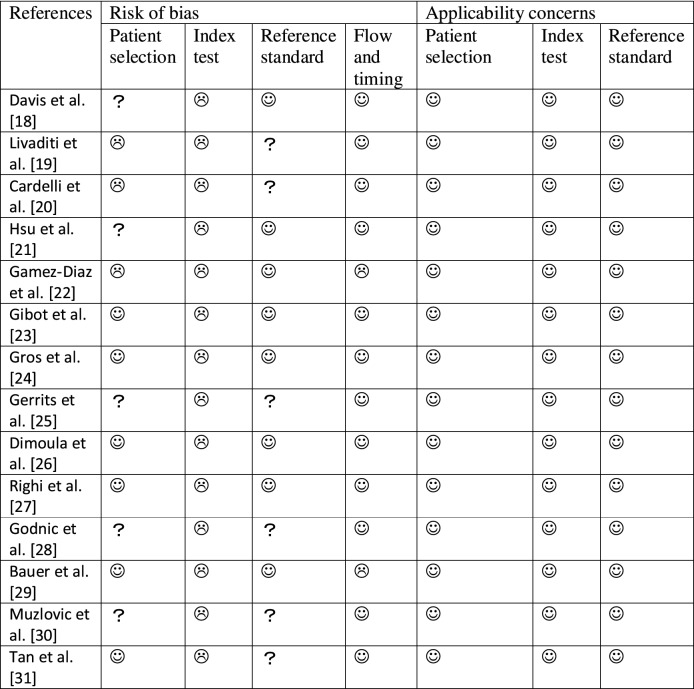
Quality assessment for 14 studies (QUADAS-2)

low risk,  high risk,  unclear risk

### Quantitative data synthesis

**Fig. 2:**
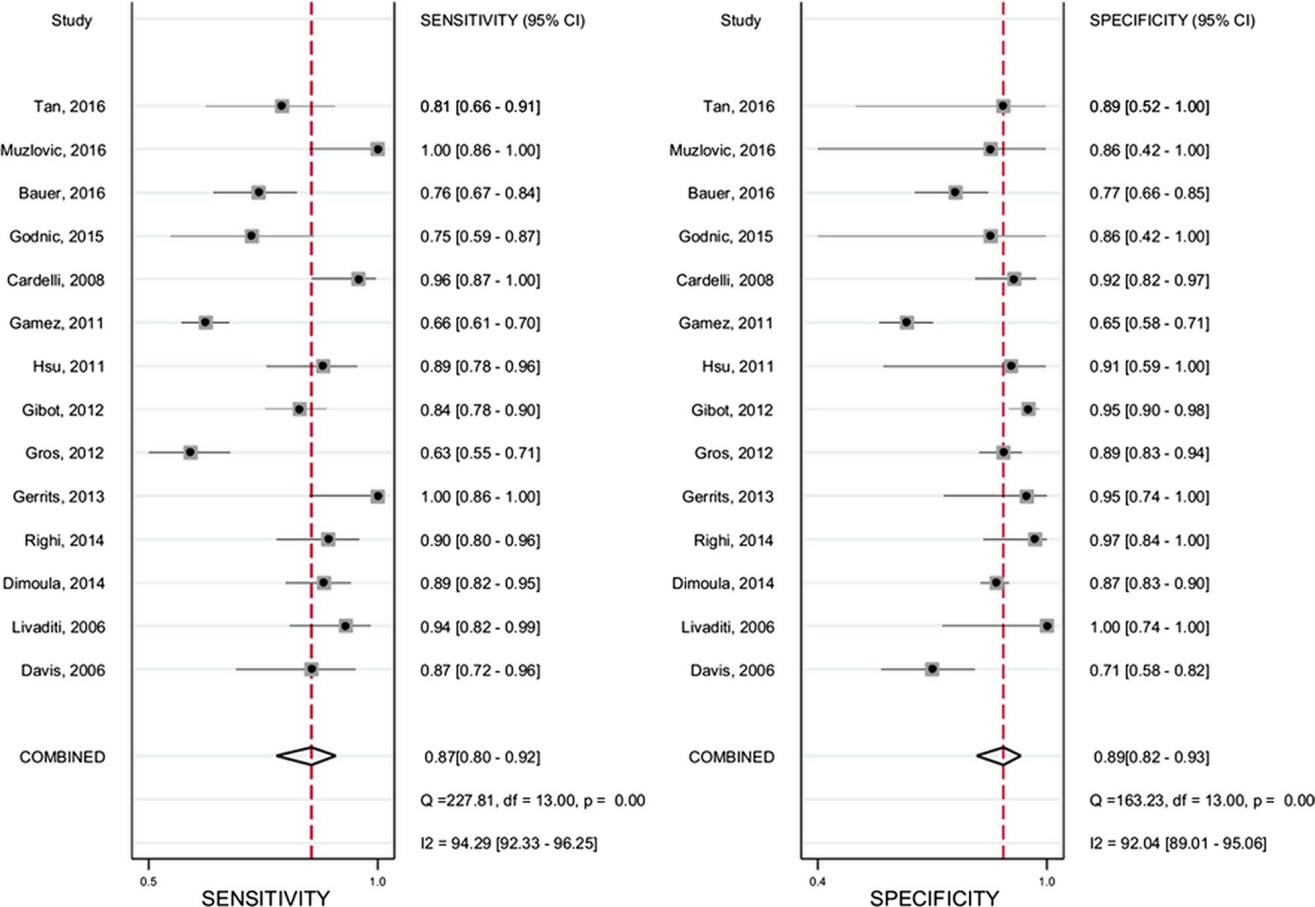
Forest plots of the sensitivity and specificity with 95% confidence interval for CD64 of 14 included studies

**Fig. 3:**
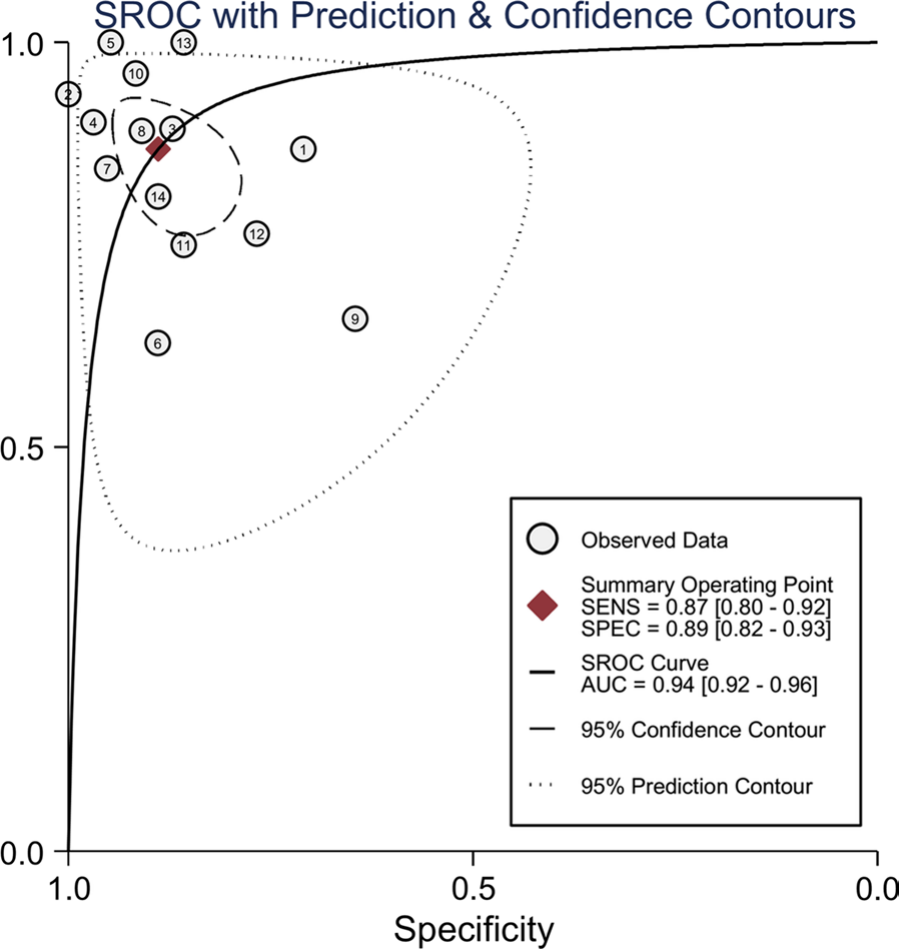
Summary receiver operator characteristic (SROC) of CD64 across 14 included studies. Each circle indicates the estimate sensitivity and specificity of each study. *AUC* area under curve, *SENS* sensitivity, *SPEC* specificity

A pooled summary of diagnostic performance revealed an adequate sensitivity of 0.87 (95% CI 0.80–0.92) and a specificity of 0.89 (95% CI 0.82–0.93) (Fig. 2). The pooled positive and negative likelihood ratios were 7.8 (95% CI 4.7–13.1) and 0.15 (95% CI 0.09–0.25), respectively. The area under the SROC curve was 0.94 (95% CI 0.92–0.96) (Fig. 3). The DOR was 53 (95% CI 22–128), indicating that the test was moderately accurate. In the 2016 sepsis consensus, “sepsis-3” was defined as organ dysfunction caused by dysregulation of the host response to infection. This definition is roughly equivalent to the definition of severe sepsis in the 2001 consensus. We measured the diagnostic accuracy of neutrophil CD64 for infection in patients with severe sepsis. Three studies (130 patients with severe sepsis/88 as a control) measured the diagnostic performance of neutrophil CD64 in patients with severe sepsis, as shown in Additional file 2: Table S1. The pooled sensitivity for these studies was 0.89 (95% CI 0.80–0.94), the pooled specificity was 0.88 (95% CI 0.56–0.98), and the pooled area under the curve was 0.92. Similar results were obtained if all patients with sepsis were taken into account.

### Heterogeneity

We assessed the nonthreshold effect, by evaluating the heterogeneity of sensitivity (Cochran’s *Q* test = 227, *P* < 0.001, *I*^2^ = 94.29) and specificity (Cochran’s *Q* test = 163.23, *P* < 0.001, *I*^2^ = 92.04). The results indicated the existence of significant heterogeneity between studies. For the threshold effect, the proportion of heterogeneity was 44%, indicating a moderate influence. However, Spearman’s correlation coefficient was 0.51 and the *P* value for this correlation analysis was 0.065, indicating that the threshold effect did not contribute to the heterogeneity between studies.

### Subgroup analysis

The results of the subgroup analysis are shown in Table 3. This analysis revealed no differences in sensitivity or specificity according to the source of patients (ICUs or EDs), assay method (in-house method or Leuko64 kit), or country of origin (in Asia or elsewhere). Studies with more than 100 patients had significantly lower pooled sensitivity (0.82 [95% CI 0.73–0.90] vs. 0.91 [95% CI 0.85–0.97], *P *< 0.01) and specificity (0.85 [95% CI 0.79–0.92] vs. 0.94 [95% CI 0.89–1.00], *P *< 0.01) values than those with fewer than 100 patients. The studies including a smaller proportion of patients with sepsis (< 50%) had a higher pooled sensitivity (0.91, 95% CI 0.86–0.95) than those with a higher proportion of patients with sepsis (> 50%) (0.85, 95% CI 0.78–0.91), and this difference was significant (*P* = 0.01).

**Table 3:** Subgroup analysis of the diagnostic accuracy of CD64 for sepsis based on univariable metaregression analysis

Subgroup	Number of studies	Pooled sensitivity (95% CI)	*P* value	Pooled specificity (95% CI)	*P* value
Source of patients					
ICUs	11	0.89 (0.83–0.94)	0.80	0.91 (0.87–0.95)	0.98
EDs	3	0.79 (0.63–0.95)		0.71 (0.58–0.84)	
Assay					
In-house FCM	9	0.88 (0.83–0.94)	0.30	0.90 (0.84–0.96)	0.27
Leuko64 kit	5	0.82 (0.70–0.94)		0.87 (0.77–0.98)	
Sample size					
Number of patients ≧ 100	7	0.82 (0.73–0.90)	< 0.01*	0.85 (0.77–0.92)	< 0.01*
Number of patients < 100	7	0.91 (0.85–0.97)		0.94 (0.89–1.00)	
Proportion of patients with sepsis					
> 50%	11	0.85 (0.78–0.91)	0.01*	0.91 (0.86–0.97)	0.87
< 50%	3	0.91 (0.86–0.95)		0.85 (0.72–0.98)	
Continent					
Asia	2	0.86 (0.71–1.00)	0.40	0.91 (0.76–1.00)	0.81
Not Asia	12	0.87 (0.81–0.93)		0.89 (0.83–0.94)	

*EDs* emergency departments, *FCM* flow cytometry, *ICUs* intensive care units

*P < 0.05

### Comparison with CRP and PCT

Six of the 14 studies directly compared the diagnostic accuracies of neutrophil CD64 and CRP [18, 26–30], as shown in Additional file 3: Table S2. The pooled sensitivity and specificity values for neutrophil CD64 were 0.84 (95% CI 0.76–0.89) and 0.81 (95% CI 0.73–0.87), and those for CRP were 0.83 (95% CI 0.78–0.86) and 0.71 (95% CI 0.56–0.85) (Table 4). A direct comparison of the two SROC curves showed the area under the curve to be greater for neutrophil CD64 than for CRP (0.89 [95% CI 0.87–0.92] vs. 0.84 [95% CI 0.80–0.88], *P *< 0.05, Additional file 4: Figure S2). Six studies directly compared neutrophil CD64 with PCT [20, 21, 23, 28–30] (Additional file 3: Table S2). The pooled sensitivity and specificity values for neutrophil CD64 were 0.83 (95% CI 0.76–0.88) and 0.88 (95% CI 0.7–0.94), and those for PCT were 0.76 (95% CI 0.61–0.86) and 0.79 (95% CI 0.70–0.86) (Table 5). The area under the SROC curve was greater for neutrophil CD64 than for PCT (0.89 [95% CI 0.84–0.95] vs. 0.79 [95% CI 0.70–0.86], *P *< 0.05, Additional file 5: Figure S3).

**Table 4:** Results from individual studies of neutrophil CD64 and C-reactive protein as markers for the diagnosis of sepsis

References	Sepsis/control (*n*)	Neutrophil CD64			C-reactive protein		
		Sensitivity	Specificity	AUC	Sensitivity	Specificity	AUC
Davis et al. [18]	38/62	0.88	0.71	–	0.88	0.59	–
Dimoula et al. [26]	103/365	0.89	0.87	0.94	0.84	0.86	0.92
Righi et al. [27]	61/32	0.90	0.97	0.93	0.89	0.41	0.71
Godnic et al. [28]	40/7	0.76	0.75	0.83	0.77	0.75	0.73
Bauer et al. [29]	110/86	0.76	0.77	0.83	0.77	0.77	0.86
Muzlovic et al. [30]	25/7	1.00	0.86	0.93	0.83	0.86	0.87
Total	377/559	0.84 (95% CI 0.76–0.89)	0.81 (95% CI 0.73–0.87)	0.89 (95% CI 0.87–0.92)	0.83 (95% CI 0.78–0.86)	0.71 (95% CI 0.56–0.85)	0.84 (95% CI 0.80–0.88)

*AUC* area under the receiver operating characteristic curve, − not available

**Table 5:** Results from individual studies of neutrophil CD64 and procalcitonin as markers for the diagnosis of sepsis

References	Sepsis/control (*n*)	Neutrophil CD64			Procalcitonin		
		Sensitivity	Specificity	AUC	Sensitivity	Specificity	AUC
Cardelli et al. [20]	52/60	0.96	0.95	0.97	0.94	0.70	–
Hsu et al. [21]	55/11	0.89	0.96	0.93	0.56	1.00	0.80
Gibot et al. [23]	154/146	0.84	0.95	0.95	0.83	0.85	0.91
Godnic et al. [28]	40/7	0.76	0.75	0.83	0.58	0.67	0.63
Bauer et al. [29]	110/86	0.76	0.77	0.83	0.73	0.74	0.82
Muzlovic et al. [30]	25/7	1.00	0.86	0.93	0.82	1.00	0.91
Total	436/317	0.83 (95% CI 0.76–0.88)	0.88 (95% CI 0.77–0.94)	0.89 (95% CI 0.84–0.95)	0.76 (95% CI 0.61–0.86)	0.79 (95% CI 0.70–0.86)	0.84 (95% CI 0.79–0.89)

*AUC* area under the receiver operating characteristic curve, − not available

### Publication bias

A *P* value of 0.05 was obtained for Deeks’ asymmetric funnel plot test, indicating a marginally significant publication bias between the studies (Additional file 6: Figure S4).

## Discussion

The early identification of sepsis remains challenging for physicians. Broad-spectrum antibiotics are widely available, but the accurate diagnosis of infection with specific biomarkers in patients with septic syndrome can reduce antibiotic use, thereby preventing the development of drug resistance. In our meta-analysis, neutrophil CD64 was found to have a good sensitivity, specificity, and AUHSROC curve (0.87, 0.90, and 0.94, respectively). The DOR was 53, indicating that neutrophil CD64 determination was a useful tool for diagnosing infection in patients with septic syndrome, based on sepsis-2 criteria, with a performance superior to that of CRP and PCT. In clinical practice, a biomarker with high sensitivity could be served as a tool to “rule out” a disease or disorder. Therefore, we believe that neutrophil CD64 owns higher sensitivity than other marker could serve as a tool to be used for patients with intermediate to lower probability of sepsis.

Neutrophil CD64 is strongly expressed on activated PMNs and can be used as a marker of bacterial infection. Only three systematic reviews and meta-analyses on the diagnostic accuracy of neutrophil CD64 for bacterial infection or sepsis have been published [9–11], two of which focused on the diagnostic accuracy of neutrophil CD64 for the diagnosis of bacterial infection [9, 10]. These two studies were heterogeneous and included adult and pediatric patients. In the study by Cid et al. [9], subgroup analysis indicated that pooled sensitivity and specificity were higher for the detection of bacterial infection in a group of adults than in a group of children (pooled sensitivity: 0.90 vs. 0.71 and pooled specificity: 0.95 vs. 0.87). In another meta-analysis performed by Li et al. [10], which included 26 studies encompassing 3944 adult and pediatric patients, CD64 was also found to be more effective in adults than in children (pooled sensitivity: 0.76 vs. 0.75 and pooled specificity: 0.87 vs. 0.82). Another meta-analysis, performed by Wang et al. [11] in 2015, included only critically ill adult patients with sepsis. In seven of the eight studies included in that meta-analysis, the patients were enrolled at ICUs, whereas the patients of the eighth study were enrolled at EDs. The study showed fair sensitivity and excellent specificity, with an excellent AUHSROC curve for the diagnosis of sepsis in critically ill adult patients (0.76 [95% CI 0.73–0.78], 0.85 [95% CI 0.82–0.87], and 0.95, respectively). By contrast, the meta-analysis reported here included more studies (11 of patients from ICUs and 3 of patients from EDs) and the sensitivity of neutrophil CD64 was found to be higher than reported by Wang et al. However, both meta-analyses revealed considerable variation in the sensitivity of neutrophil CD64. In the meta-analysis performed by Wang et al., there was also heterogeneity due to a nonthreshold effect, but the source of heterogeneity was not examined further. We found that the heterogeneity between studies was associated with sample size and with the proportion of patients with sepsis enrolled in the study.

CRP and PCT are the biomarkers most widely used in screening of infection in septic patients. In our study, the CRP and PCT showed similar area under SROC (0.84 vs. 0.84). However, because different studies were included in the CRP group and PCT group, we cannot compare the result directly. PCT is often reported to perform better than CRP as a maker of sepsis [32]. In a meta-analysis performed in 2006 by Uzzan et al. [33], the DORs of PCT and CRP were 15.7 and 5.4, respectively. Based on these findings, new biomarkers are required to improve the clinical management of sepsis. Our previous meta-analysis showed presepsin to have a diagnostic performance equivalent to those of CRP and PCT [34]. We show here that neutrophil CD64 has a performance superior to those of PCT and CRP, with a higher area under the HSROC curve. Neutrophil CD64 may therefore be considered a superior candidate biomarker.

We conducted a subgroup analysis to compare the results obtained with the Leuko64 kit and in-house flow cytometry. We found no difference in sensitivity and specificity results between these two methods. The Leuko64 kit used premixed fluorescein-labeled anti-CD64 and anti-CD163 monoclonal antibodies, and Leuko64 software was used for the final analysis. The results are expressed as the Leuko64 index, calculated as the ratio of the mean fluorescence intensity of the cell population to that of the beads. In a meta-analysis published by Li et al. [10] examining the use of neutrophil CD64 for the diagnosis of bacterial infection, the subgroup analysis showed sensitivity and specificity to be higher for in-house flow cytometry than for the Leuko64 kit. This difference in subgroup analysis may be due to the inclusion of different studies. A standardized platform for neutrophil CD64 is therefore required, to assist clinical laboratory and physicians in the diagnosis of sepsis.

The gold standard for identification of sepsis in the included studies was based on the 2001 criteria (sepsis-2) [5]. These criteria define sepsis as a systemic inflammatory response syndrome (SIRS), together with a known or suspected infection. All the studies included in our meta-analysis used these criteria as a reference standard for the diagnosis of sepsis. Despite the limited sensitivity and specificity of SIRS for the detection of sepsis [35], these criteria have been widely used in sepsis studies. The Society of Critical Care Medicine and the European Society of Intensive Care Medicine recently proposed a new definition of sepsis based on the change in sequential organ failure assessment score in patients with a known or suspected infection (sepsis-3) [4]. No studies on the use of neutrophil CD64 for diagnosing sepsis based on the sepsis-3 criteria have been published as yet. We found three studies analyzing the diagnostic performance of neutrophil CD64 in patients with severe sepsis based on the sepsis-2 criteria, with the pooled result showing sufficient sensitivity and specificity. Further studies are required to assess the diagnostic performance of neutrophil CD64 as a biomarker for infection in patients with septic syndrome, based on the sepsis-3 criteria.

The timing of measurement is related to the diagnostic accuracy of biomarkers. In vitro study showed that neutrophil CD64 increases within 10–120 min after exposing to lipopolysaccharide and can be stable for over 48 h [36]. Another study performed by Ng et al. showed neutrophil CD64 had high sensitivities (87–97%) and specificities (88–90%) during 48 h after sepsis onset [37]. Serial testing of neutrophil CD64 also showed to be good monitor of appropriate antibiotic treatment [26].

To accurately identify the septic patients, we require a high sensitivity and specificity biomarker to reduce the diagnosis uncertainty. Widely used SIRS criteria is insufficiently sensitive and specific [38, 39]. A clinical diagnostic test with high sensitivity and low specificity will yield large number of false positives and cause unnecessary antibiotic use. On the other hand, a clinical diagnostic test with low sensitivity and high specificity (e.g., microbial culture) will cause missed diagnosis. Thus, combined different biomarkers may improve the sepsis diagnosis. Two studies included in our meta-analysis showed that combination of different biomarkers showed superior performance than single biomarker alone.  The study conducted by Gibot et al. addressed the benefit of combination of PCT, sTREM-1, and neutrophil CD64 had better performance than each individual biomarker [23]. Another study, performed by Bauer et al., showed model that included CRP, PCT, and neutrophil CD64 performed better than CRP alone (AUC 0.90 vs. 0.86, *P* = 0.03) [29]. Further studies are needed to test which combination is ideal for sepsis diagnosis.

Sepsis can be caused by different types of pathogens, including bacteria, fungus, and virus. Previous studies have shown that neutrophil CD64 is promising in diagnosing bacterial sepsis, but not in fungal and viral infections [9, 10]. One study performed by Righi et al. compared the bacterial and fungal infections with neutrophil CD64 expression level and did not show any significant difference, but the patient number is small in the study [27]. Further studies to determine the accuracy of neutrophil CD64 in fungal or viral sepsis are required.

This study has several limitations. First, no predefined specific cutoff criteria for neutrophil CD64 were used in any of the studies included. The effect of testing may, therefore, be overestimated. The optimal cutoff value for neutrophil CD64 is currently unknown and warrants further investigation. Second, the number of studies comparing neutrophil CD64 with CRP or PCT was small. More studies are required to confirm the findings of our meta-analysis. Third, some of the studies included had small sample sizes, resulting in heterogeneity in the meta-analysis.

## Conclusions

In conclusion, in this meta-analysis of data from 14 studies including 2471 patients, neutrophil CD64 level was found to be an effective diagnostic biomarker for infection in patients with septic syndrome based on sepsis-2 criteria. Further analysis showed that neutrophil CD64 outperformed CRP and PCT. Additional studies are required to confirm that neutrophil CD64 level is an effective biomarker for diagnosing infection in patients with septic syndrome, based on sepsis-3 criteria.

## Additional files

**Additional file 1: Figure S1.** Methodological quality of the 14 studies included, according to QUADAS-2.

**Additional file 2: Table S1.** Summary of diagnostic accuracy data for neutrophil CD64 in patients with severe sepsis, for the studies included.

**Additional file 3: Table S2.** Summary of diagnostic accuracy for biomarkers, neutrophil CD64, procalcitonin, and C-reactive protein, for the studies included.

**Additional file 4: Figure S2.** Comparison of summary receiver operating characteristic curves between neutrophil CD64 (○) and C-reactive protein (CRP) (◇) for the diagnosis of sepsis. The p value was < 0.05.

**Additional file 5: Figure S3.** Comparison of summary receiver operating characteristic curves between neutrophil CD64 (○) and procalcitonin (PCT) (△) for the diagnosis of sepsis. The p value was < 0.05.

**Additional file 6: Figure S4.** Deeks’ funnel plot asymmetry test for publication bias. A marginally significant publication bias was found between studies (p = 0.05). ESS = effective sample size.

## Abbreviations

AUHSROC curve: area under the summary receiver operating characteristic curve
CD64: cluster of differentiation 64
CI: confidence interval
CRP: C-reactive protein
DOR: diagnostic odds ratio
EDs: emergency departments
HSROC: hierarchical summary receiver operating characteristic
ICUs: intensive care units
PCT: procalcitonin
PMNs: polymorphonuclear leukocytes
QUADAS-2: Quality Assessment of Diagnostic Accuracy Studies-2
SIRS: systemic inflammatory response syndrome
SOFA: sequential organ failure assessment
SROC: summary receiver operating characteristic
