# Supplementary figures and images for: Comparison of the accuracy of neutrophil CD64, procalcitonin, and C-reactive protein for sepsis identification: a systematic review and meta-analysis
Source: Ann Intensive Care. 2019 Jan 8;9:5. doi: 10.1186/s13613-018-0479-2 (PMC6325056; doi:10.1186/s13613-018-0479-2)

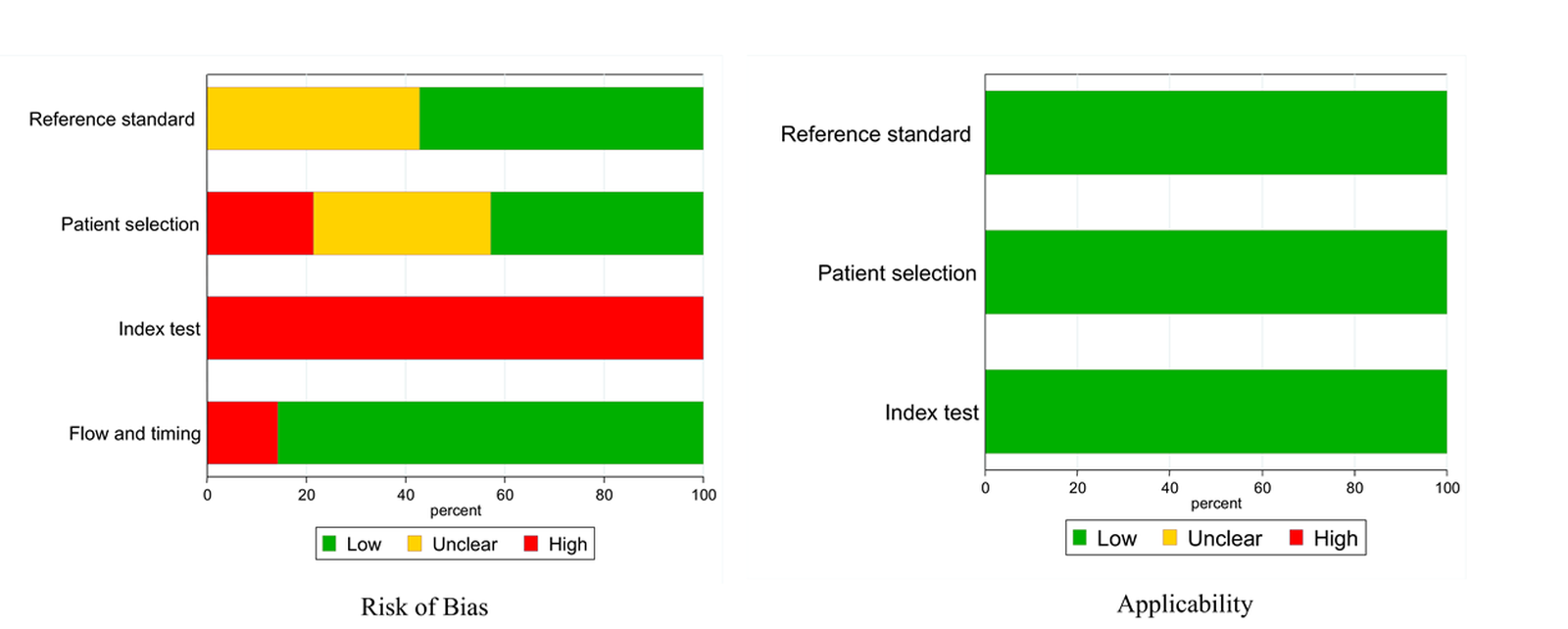

Supplement: Supplementary file 1 — Additional file 1: Figure S1. Methodological quality of the 14 studies included, according to QUADAS-2. [file 13613_2018_479_MOESM1_ESM.tif]

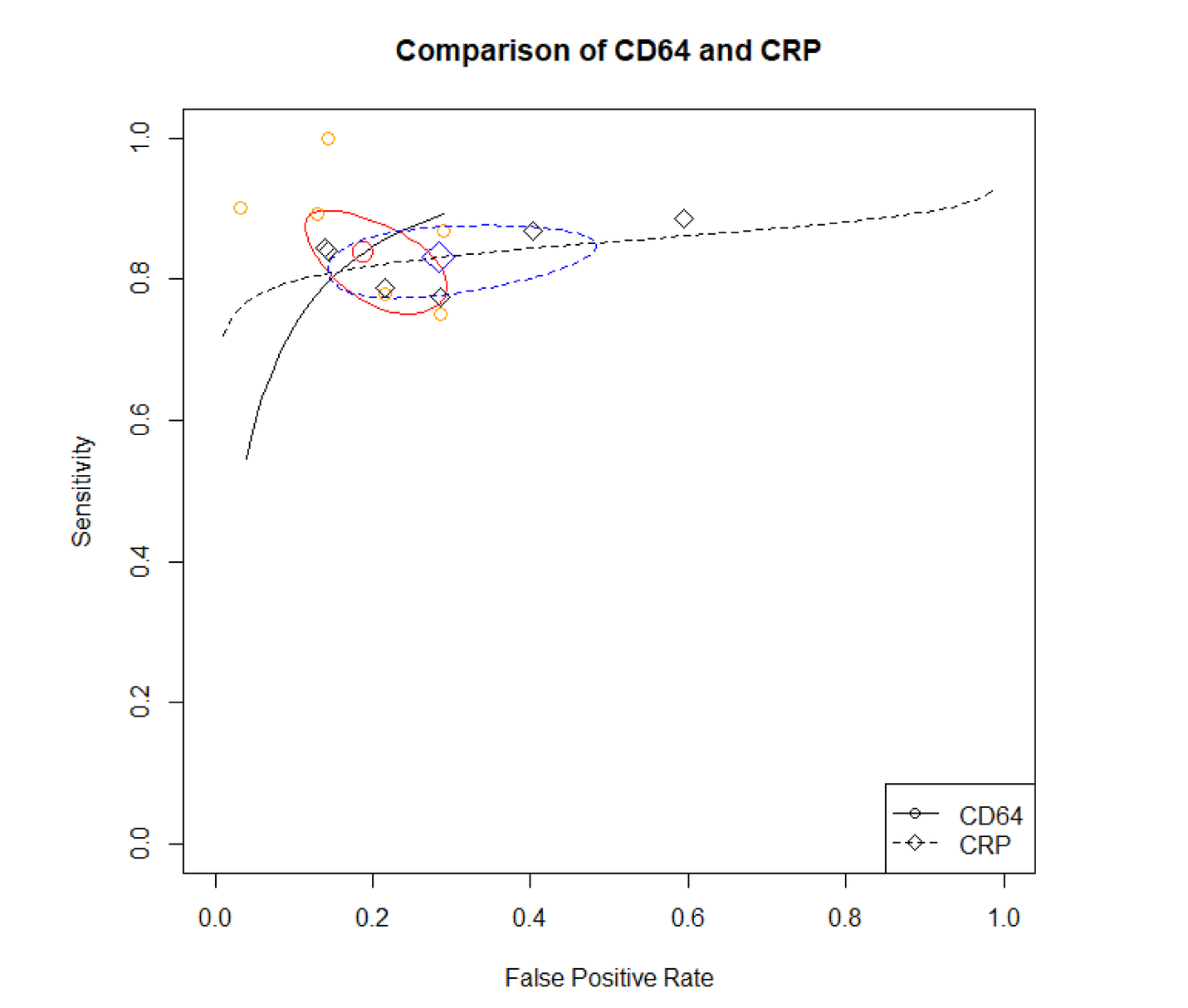

Supplement: Supplementary file 4 — Additional file 4: Figure S2. Comparison of summary receiver operating characteristic curves between neutrophil CD64 (○) and C-reactive protein (CRP) (◇) for the diagnosis of sepsis. The p value was < 0.05. [file 13613_2018_479_MOESM4_ESM.tif]

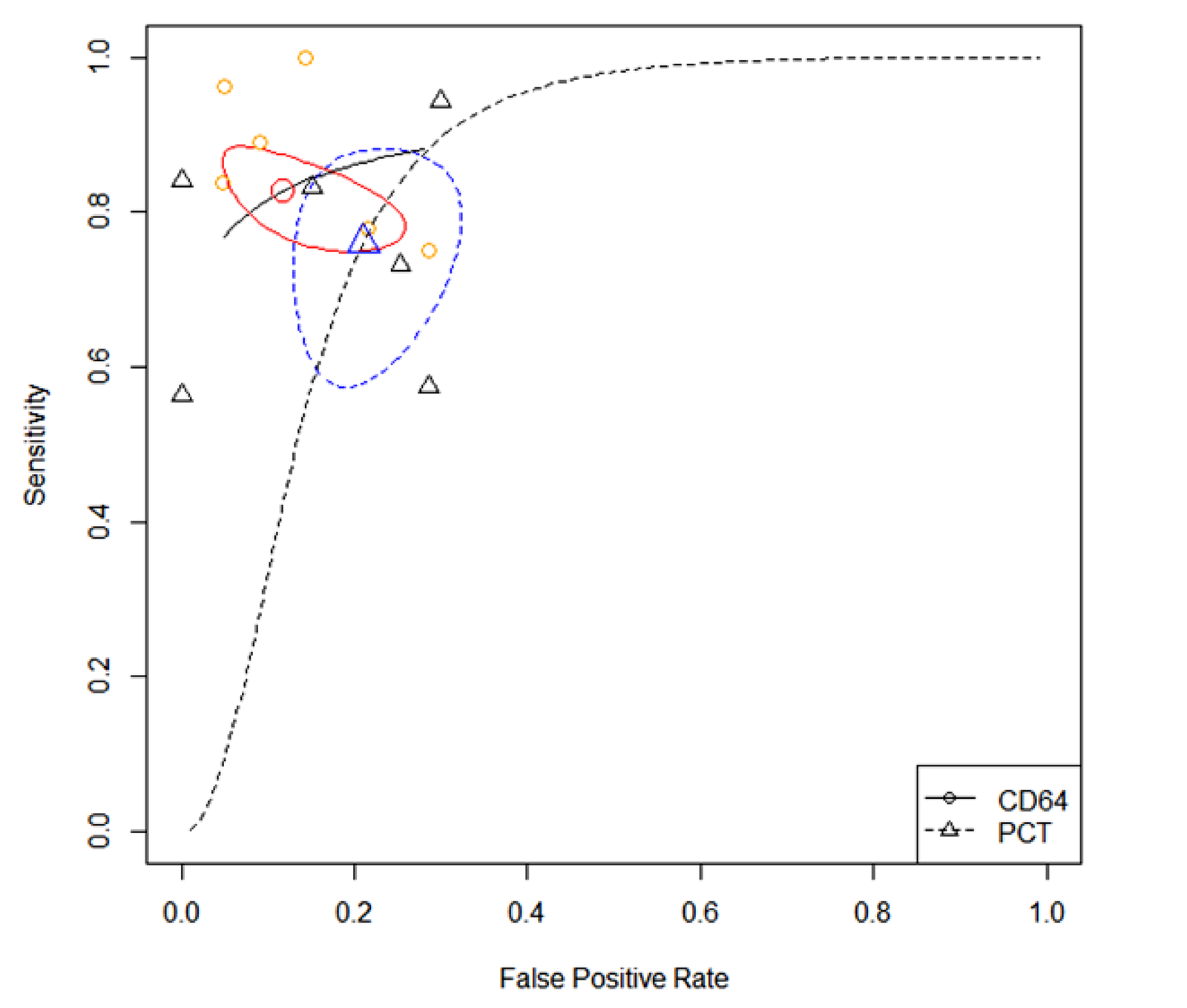

Supplement: Supplementary file 5 — Additional file 5: Figure S3. Comparison of summary receiver operating characteristic curves between neutrophil CD64 (○) and procalcitonin (PCT) (△) for the diagnosis of sepsis. The p value was < 0.05. [file 13613_2018_479_MOESM5_ESM.tif]

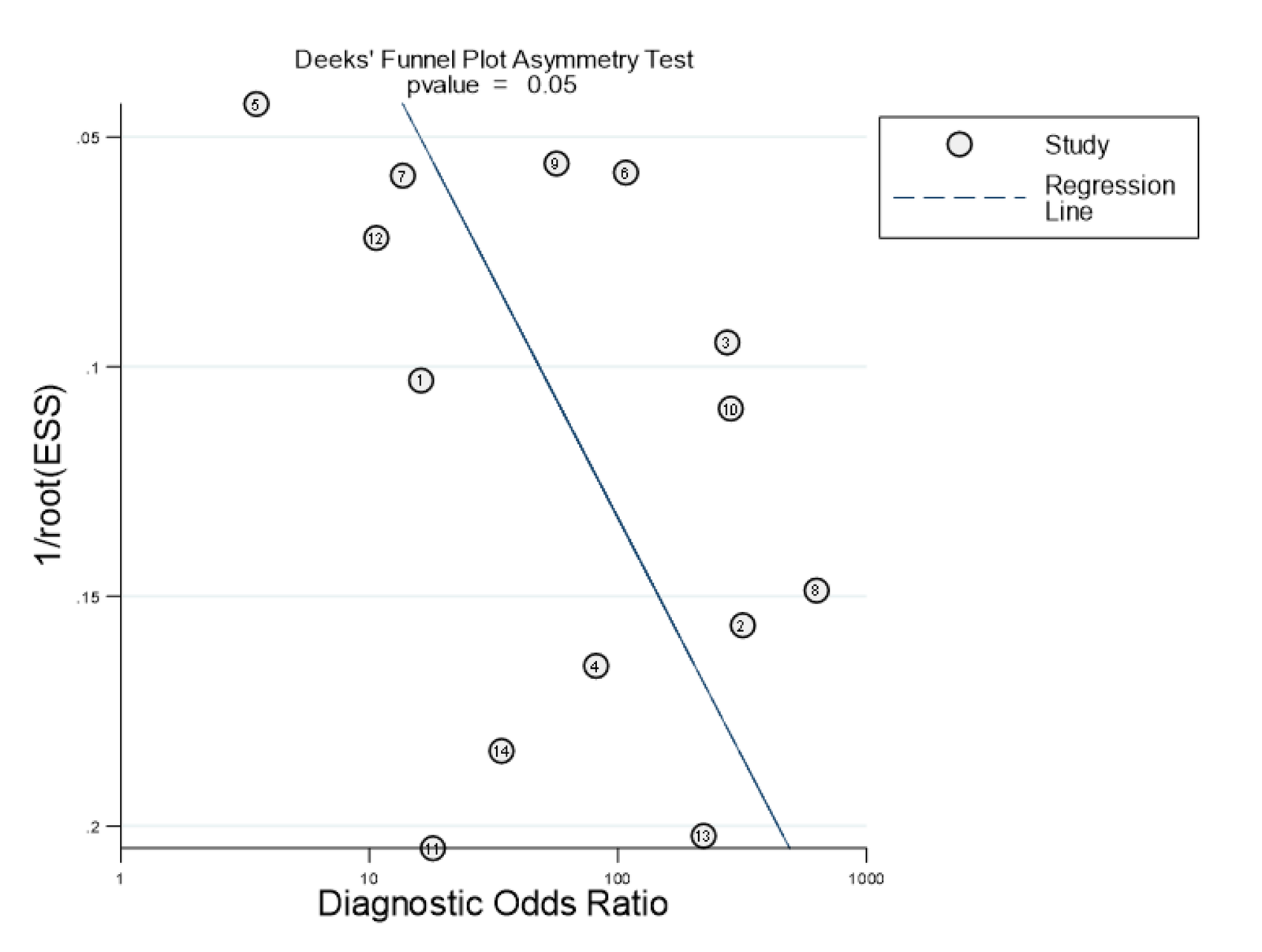

Supplement: Supplementary file 6 — Additional file 6: Figure S4. Deeks’ funnel plot asymmetry test for publication bias. A marginally significant publication bias was found between studies (p = 0.05). ESS = effective sample size. [file 13613_2018_479_MOESM6_ESM.tif]
