# Supplementary material for: Comparison of the accuracy of neutrophil CD64, procalcitonin, and C-reactive protein for sepsis identification: a systematic review and meta-analysis
Source: Ann Intensive Care. 2019 Jan 8;9:5. doi: 10.1186/s13613-018-0479-2 (PMC6325056; doi:10.1186/s13613-018-0479-2)
Supplement: Supplementary file 2 — Additional file 2: Table S1. Summary of diagnostic accuracy data for neutrophil CD64 in patients with severe sepsis, for the studies included. [file 13613_2018_479_MOESM2_ESM.docx]

Table S1: Summary of diagnostic accuracy data for neutrophil CD64 in patients with severe sepsis, for the studies included.

| Author, year | Severe sepsis/  control (n) | CD64 | | |
| --- | --- | --- | --- | --- |
|  |  | Sensitivity | Specificity | AUC |
| Livaditi (2006)[19] | 47/12 | 0.95 | 1.00 | 0.98 |
| Hsu (2011)[21] | 55/11 | 0.89 | 0.96 | 0.93 |
| Righi (2014) [27] | 28/65 | 0.86 | 0.70 | - |
| Total | 130/88 | 0.89 (95% CI: 0.80-0.94) | 0.88 (95% CI: 0.56-0.98) | 0.92 |

AUC = area under the receiver operating characteristic curve; − = Not available
