# Supplementary material for: Comparison of the accuracy of neutrophil CD64, procalcitonin, and C-reactive protein for sepsis identification: a systematic review and meta-analysis
Source: Ann Intensive Care. 2019 Jan 8;9:5. doi: 10.1186/s13613-018-0479-2 (PMC6325056; doi:10.1186/s13613-018-0479-2)
Supplement: Supplementary file 3 — Additional file 3: Table S2. Summary of diagnostic accuracy for biomarkers, neutrophil CD64, procalcitonin, and C-reactive protein, for the studies included. [file 13613_2018_479_MOESM3_ESM.docx]

Table S2: Summary of diagnostic accuracy for biomarkers, neutrophil CD64, procalcitonin, and C-reactive protein, for the studies included

| Author, year | Sepsis/  control (n) | CD64 | | | Procalcitonin | | | C-reactive protein | | |
| --- | --- | --- | --- | --- | --- | --- | --- | --- | --- | --- |
|  |  | Sensitivity | Specificity | AUC | Sensitivity | Specificity | AUC | Sensitivity | Specificity | AUC |
| Davis (2006)[18] | 38/62 | 0.88 | 0.71 | - | - | - | - | 0.88 | 0.59 | - |
| Livaditi (2006)[19] | 47/12 | 0.95 | 1.00 | 0.98 | - | - | - | - | - | - |
| Cardelli (2008)[20] | 52/60 | 0.96 | 0.95 | 0.97 | 0.94 | 0.70 | - | - | - | - |
| Hsu (2011)[21] | 55/11 | 0.89 | 0.96 | 0.93 | 0.56 | 1.00 | 0.80 | - | - | - |
| Gamez (2011)[22] | 404/206 | 0.66 | 0.65 | 0.71 | - | - | - | - | - | - |
| Gibot (2012)[23] | 154/146 | 0.84 | 0.95 | 0.95 | 0.83 | 0.85 | 0.91 | - | - | - |
| Gros (2012)[24] | 148/145 | 0.63 | 0.89 | 0.80 | - | - | - | - | - | 0.78 |
| Gerrits (2013 [25] | 25/19 | 1.00 | 0.95 | - | - | - | - | 1.00 | - | - |
| Dimoula (2014)[26] | 103/365 | 0.89 | 0.87 | 0.94 | - | - | - | 0.84 | 0.86 | 0.92 |
| Rhghi (2014)[27] | 61/32 | 0.90 | 0.97 | 0.93 | - | - | - | 0.89 | 0.41 | 0.71 |
| Godnic (2015) [28] | 40/7 | 0.76 | 0.75 | 0.83 | 0.58 | 0.67 | 0.63 | 0.77 | 0.75 | 0.73 |
| Bauer (2016)[29] | 108/79 | 0.78 | 0.79 | 0.85 | 0.73 | 0.75 | 0.82 | 0.79 | 0.79 | 0.86 |
| Muzlovic (2016)[30] | 25/7 | 1.00 | 0.86 | 0.93 | 0.82 | 1.00 | 0.91 | 0.83 | 0.86 | 0.87 |
| Tan (2016)[31] | 42/9 | 0.81 | 0.89 | 0.88 | - | - | - | - | - | - |

AUC = area under the receiver operating characteristic curve; − = Not available
